# Behavior Assessment in Children Following Hospital-Based General Anesthesia versus Office-Based General Anesthesia

**DOI:** 10.3390/dj4030027

**Published:** 2016-08-15

**Authors:** LaQuia A. Vinson, Matthew L. Rasche, Brian J. Sanders, James E. Jones, Mark A. Saxen, Angela M. Tomlin, James A. Weddell

**Affiliations:** 1 Department of Pediatric Dentistry Indiana University School of Dentistry, James Whitcomb Riley Hospital for Children Indianapolis, Indiana, IN 46202, USA; bjsander@iu.edu (B.J.S.); jej7@iu.edu (J.E.J.); jweddell@iu.edu (J.A.W.); 2 Private Practice, Bloomington, IN 47401, USA; mrasche@gmail.com; 3 Department of Oral Pathology, Medicine, and Radiology, Indiana University School of Dentistry Indianapolis, Indiana, IN 46202, USA; msaxen93@gmail.com; 4 Department of Pediatrics Indiana University School of Medicine, James Whitcomb Riley Hospital for Children Indianapolis, Indiana, IN 46202, USA; atomlin@iu.edu

**Keywords:** behavior management, hospital denistry, general anesthesia, infant oral health, early childhood caries

## Abstract

The purpose of this study was to determine if differences in behavior exist following dental treatment under hospital-based general anesthesia (HBGA) or office-based general anesthesia (OBGA) in the percentage of patients exhibiting positive behavior and in the mean Frankl scores at recall visits. This retrospective study examined records of a pediatric dental office over a 4 year period. Patients presenting before 48 months of age for an initial exam who were diagnosed with early childhood caries were included in the study. Following an initial exam, patients were treated under HBGA or OBGA. Patients were followed to determine their behavior at 6-, 12- and 18-month recall appointments. Fifty-four patients received treatment under HBGA and 26 were treated under OBGA. OBGA patients were significantly more likely to exhibit positive behavior at the 6- and 12-month recall visits (*p* = 0.038 & *p* = 0.029). Clinicians should consider future behavior when determining general anesthesia treatment modalities in children with early childhood caries presenting to their office.

## 1. Introduction

Administration of general anesthesia has historically served an important role in the practice of dentistry by allowing specific patient populations such as young children greater access to comprehensive dental care [1,2]. In recent years, behavior management techniques used by pediatric dentists have evolved toward increased use of general anesthesia in accordance with changing parenting philosophies, expectations, and safe outcomes [3]. Parents have become more willing to accept dental treatment of their children using general anesthesia over other behavior management techniques [4]. The use of general anesthesia allows both the practitioner and patient a modality in which safe, comprehensive treatment can be performed under optimal conditions [5,6,7]. It has been suggested that treatment performed under general anesthesia is of higher quality than treatment performed during other forms of behavior management [8]. However, rising costs and scheduling difficulties of traditional hospital-based general anesthesia (HBGA) services have resulted in increased use of office-based general anesthesia (OBGA) services for dental treatment [5,9,10].

The American Dental Association’s guidelines on the use of sedation and general anesthesia by dentists define general anesthesia as a drug-induced loss of consciousness during which patients cannot be aroused, even from painful stimulation [2]. General anesthesia can be obtained through different pharmacologic routes of administration while hospital-based general anesthesia (HBGA) is obtained primarily from the administration of inhalation medications. It is common for office-based general anesthesia (OBGA) to be obtained primarily from parenteral intravenous drug administration.

Classifying pediatric dental patients’ behavior has been historically reported using the Frankl method of classification, and managing these patients with sedation is widely accepted among dental practitioners [11]. A number of studies have reported on patients’ behavior during subsequent recall visits following different forms of sedation. Kupietzky and Blumenstyk reported data using the Frankl scale on behavior of patients following treatment under general anesthesia versus oral conscious sedation [12]. The 1998 study reported no difference in patients’ behavior at subsequent recall visits between patients treated under conscious sedation versus general anesthesia for children receiving dental treatment at a young age. O’Sullivan et al. performed a retrospective study of 80 children treated under general anesthesia that were followed for at least 2 years post treatment [13]. Of the children requiring additional restorative dental treatment, 80% readily accepted dental care using local anesthesia without the assistance of conscious or general sedation techniques. Peretz et al. evaluated children receiving dental treatment under conscious sedation and general anesthesia from the Hebrew University-Hadassah School of Medicine pediatric dental clinic. They reported similar behavior between the two groups as evidenced by Frankl scores 13 months postoperatively between children that had been treated for early childhood caries (ECC) by the use of oral conscious sedation or general anesthesia [14].

Fuhrer et al. reported on patient behavior during three subsequent recall visits following dental treatment rendered under conscious sedation and general anesthesia in 80 patients from a pediatric dental office [15]. Patients included those who presented before 36 months of age with ECC for an initial exam. Following treatment, patients were followed to determine behavior at 6-, 12-, and 18-month preventive recall appointments. Positive behavior was defined as a Frankl score of 3 or 4. They concluded that patients who were treated under general anesthesia, as opposed to conscious sedation, were approximately four times more likely to exhibit positive behavior at the 6 month recall appointment. Behavior at 12- and 18-month recall appointments, although not statistically significant, trended towards positive behavior. They also concluded that clinicians should consider future behavior when determining treatment modalities for children.

Treatment performed under general anesthesia has been shown to have many benefits for both the patient and dental team [1,7,8,13]. Currently, there have been no studies that have evaluated future behavior after treatment of patients with hospital based general anesthesia versus office-based general anesthesia. The purpose of the present study was to assess behavior and determine if there were differences in the percentage of patients exhibiting positive behavior following hospital based general anesthesia (HBGA) versus office based general anesthesia (OBGA).

## 2. Materials and Methods

This retrospective study was approved by the Institutional Review Board of Indiana University (Study #EX1002-16). Records of patients who received dental treatment under HBGA and OBGA from a pediatric dental office over a four-year period were reviewed. Inclusion criteria included patients who presented to the pediatric dental office for an oral exam and were diagnosed with early childhood caries (ECC). Early childhood caries is defined by the American Academy of Pediatric Dentistry (AAPD) as a patient having at least 1 carious lesion, 1 tooth missing due to caries or 1 dental restoration prior to 48 months of age [16]. Information was collected from the patient’s clinical notes concerning age, race, gender, behavior at the initial and recall exams, and existing medical conditions of each patient. Patients who had medical conditions that could potentially influence behavior were included. The Frankl behavior scale was used to assess patient’s behavior in this investigation [17] (Table 1).

A retrospective chart review was performed of the patients’ previously recorded behavior. Behavior scores were recorded by one of the three pediatric dentists who had examined the patient at the time of their initial visit and were determined to be a 1, 2, 3 or 4 according to the Frankl scale. Positive Behavior was defined as a Frankl Score of 3 or 4. In this study, the decision to use one form of pharmacologic behavior management over another was not a randomized assignment: the decision to use either method was determined by the pediatric dentist who examined the patient at their initial visit. To account for possible biases in the data between the two groups, Frankl score baseline behavior, comorbidities (asthma, sensory disabilities, heart murmurs, etc.), age, gender, race, severity of dental problems, and all other collected factors were examined and adjusted for in the analyses. For example, if most children with autism were given HBGA then the analyses needed to account for autism since there may be differences in behavior between children with or without autism.

The patients treated under HBGA all received care at a pediatric outpatient surgery center under the supervision of a pediatric anesthesiologist. The patients treated under OBGA all received care at a dental office under the supervision of a dentist anesthesiologist. Patients in both groups were treated and released with no unexpected outcomes. The patients’ Frankl scores were recorded following treatment under HBGA or OBGA at the 6-, 12-, and 18-month preventive recall appointments. Groups were tested for differences in the percentages exhibiting positive behavior at the 6-, 12-, and 18-month recall appointments.

The HBGA and OBGA groups were compared for differences in age, number of treated teeth, and initial-visit Frankl score using two-sample *t*-tests and for differences in sex, race, presence of any comorbidities, presence of asthma, and initial-visit positive behavior (Frankl score 3 or 4) using chi-square tests. Associations of subsequent 6-, 12-, and 18-month recall visits, Frankl scores with age, number of treated teeth, and initial visit Frankl scores were evaluated using correlation coefficients and with each anesthesia group, sex, race, and presence of any comorbidities using one-way ANOVA. Associations of recall-visit positive behavior with age and number of treated teeth were evaluated using two-sample *t*-tests and with each anesthesia group, sex, race, and presence of any comorbidities using chi-square tests. Multiple-variable analyses of Frankl scores were performed using analysis of covariance, while multiple-variable analyses of positive behavior were performed using logistic regression.

## 3. Results

Fifty-four patients fitting the inclusion criteria treated under HBGA were identified and 26 were identified as treated under OBGA. Subject characteristics are shown in (Table 2).

The hospital-based general anesthesia group had significantly more treated teeth than the office-based general anesthesia group (*p* = 0.002). There were no other statistically significant differences between the two groups at the initial visit. Behavior at the initial exam showed a relationship with behavior at the recall exams, so behavior at the initial exam was included as a covariate in the analyses comparing the behaviors between anesthesia groups. No other variables were significantly associated with positive behavior or Frankl scores at the recall visits.

**Figure 1:**
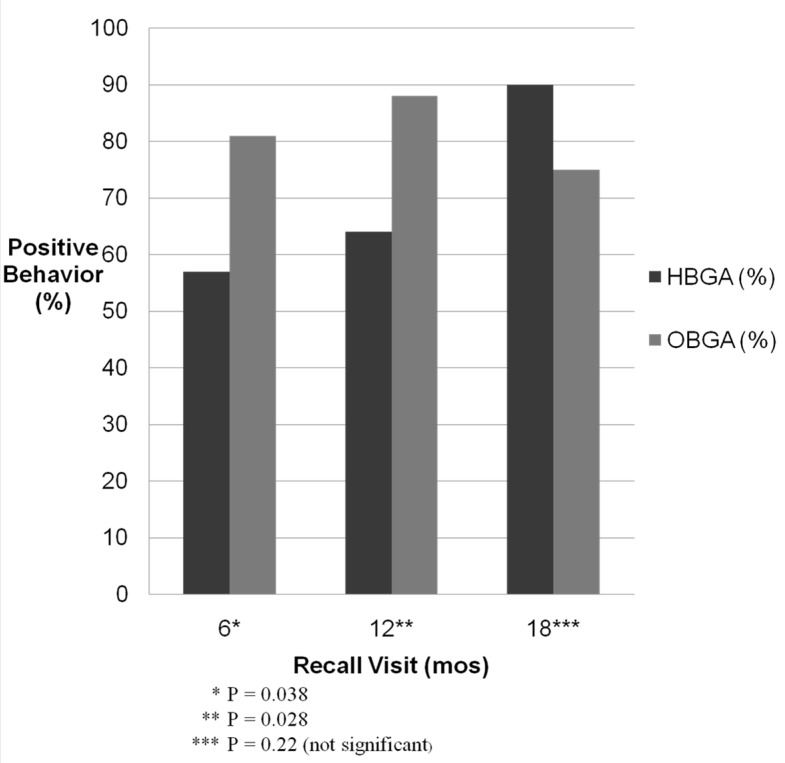
Percentage of postive behavior at follow up visits.

The associations of anesthesia administration with the behavior outcomes were analyzed using multiple-variable models that included number of treated teeth and behavior at the initial exam as covariates. A significantly higher proportion of OBGA patients had positive behavior than HBGA patients at the six-month recall visit (*p* = 0.038) and 12-month recall visit (*p* = 0.028) (Figure 1).

The difference was not statistically significant at the eighteen-month recall visit (*p* = 0.22). The mean Frankl scores were significantly higher for the HBGA patients than for the OBGA patients at the eighteen-month recall visit (*p* = 0.019) (Table 3).

## 4. Discussion

In this study, the majority of the patients exhibited baseline behavior that was classified as either definitely negative to negative at the initial visit. To minimize influences of prior dental experiences, this study included patients who were selected before the age of 48 months. Children in this age group would be most likely to have positively affected behavior at follow-up visits. In this study, patients were more likely to exhibit positive behavior following dental treatment for ECC under OBGA than HBGA at their 6- and 12-month recall appointments. The trend toward a higher Frankl score at subsequent recall visits also follows a natural progression of young patients to exhibit improved behavior over time as they grow and mature.

A significant dropout rate occurred between the 6- and 12-month to the 18-month recall appointments. This may lead to careful interpretation of the 18-month data as there are a large number of subjects without this data. The Frankl score means at 6- and 12-months showed that the HBGA had lower Frankl scores at these exams but higher scores at 18-months. This may be a non-significant trend as the tests were not statistically significant. Unfortunately, disparity in maintaining routine dental visits is a consistent finding among patients of lower socioeconomic status, which may contribute to the dropout rate that was seen in this study [18].

O’Sullivan’s retrospective study of 80 children treated under general anesthesia with greater than 2 year follow-up appointments revealed that patients readily accepted dental care chair side using local anesthesia [13]. The present study revealed an increase in mean Frankl score at follow-up appointments with both general anesthesia groups that may be consistent with findings from the O’Sullivan study. Although there was not a large sample size, the findings of this study suggest there was a difference in behavior within the general anesthesia group as patients in the OBGA group were more likely to exhibit positive behavior than patients in the HBGA group at 6- and 12-month recall appointments. Nonetheless, this likelihood of future positive behavior may influence a clinician and parent to be more apt to select OBGA over HBGA for dental treatment.

By their nature, retrospective studies can portray an inherent amount of bias associated with their findings. An attempt was made to limit bias by creating a tailored study design and using specific inclusion criteria. In this study patients were seen by different practitioners at their appointments, which may have limited the consistency of recorded behavior due to different interpretations of the Frankl scale. All patients treated under OBGA received care in the same office from the same dental anesthesiologist to control the protocol and limit bias of the retrospective study. Patients treated under HBGA were all treated in the same outpatient setting under the care of a pediatric anesthesiologist consistent with the standard hospital protocols. As a retrospective study, it was not possible to create a “blind” environment for the data collector. A larger sample size may be warranted necessary to draw stronger conclusions and future studies may be needed to reveal potential influences of comorbidities or other differences between each groups’ behavior following general anesthesia.

Advanced pharmacologic behavioral management techniques utilizing general anesthesia have received increased acceptance in contemporary society concurrent with changes in parenting practices [3,4]. Dentists perceive that children have worse behavior due to changes in parenting [3]. Even so, hospital-based general anesthesia has traditionally filled an important niche in pediatric dentistry. Office-based general anesthesia has emerged in recent years as a valuable and safe alternative approach for dental treatment [5,9,10]. Office-based general anesthesia may allow for increased convenience, ease of scheduling, and cost savings as compared to hospital-based general anesthesia for both patients and dentists [5]. The future of OBGA appears robust as organized dentistry and state legislatures react to economic conditions and public demands for safe, convenient cost-effective anesthesia care [9,19,20].

## 5. Conclusions

1. Pediatric dental patients were more likely to exhibit positive behavior at the 6- and 12-month recall appointments following dental treatment for early childhood caries under OBGA than HBGA.
2. Clinicians may consider future behavior of dental patients when determining general anesthesia treatment modalities in children with early childhood caries presenting to their office.

## Figures and Tables

**Table 1:** Definition of the Frankl Scale.

Frankl Scale	Rating	Description
Definitely Negative	1	Refusal of treatment, forceful crying, fearfulness, or any other overt evidence of extreme negativism.
Negative	2	Reluctance to accept treatment, uncooperativeness, some evidence of negative attitude but not pronounced (sullen, withdrawn).
Positive	3	Acceptance of treatment; cautious behavior at times; willingness to comply with the dentist, at times with reservation, but patient follows the dentist’s direction cooperatively.
Definitely Positive	4	Good rapport with the dentist, interest in the dental procedures, laughter and enjoyment.

**Table 2:** Subject characteristics and demographics.

Category	HBGA, N (%)	OBGA
Male	33 (61)	14 (54)
Female	21 (39)	12 (46)
Total	54	26
African American	17 (31)	13 (50)
Asian	2 (4)	1 (4)
Caucasian	12 (22)	6 (23)
Hispanic Origin	23 (43)	6 (23)
Age, mean ± SD	2.8 ± 0.8	3.0 ± 0.7
Asthma	7 (13)	2 (8)
Any Comorbidity	11 (20)	4 (15)
# Treated Teeth, mean ± SD	10.8 ± 3.6	8.2 ± 3.0

**Table 3:** Mean Frankl scores at follow-up visits.

Recall Appointment Interval	HBGA Mean (SD)	OBGA Mean (SD)	*p*-Value
6 months	2.5 (1.2)	3.0 (0.8)	*p* = 0.19
12 months	2.9 (1.2)	3.4 (1.0)	*p* = 0.08
18 months *	3.4 (0.9)	2.9 (1.2)	*p* = 0.019

* Data is statistically significant (*p* = 0.019). Due to large number of subjects without this data, it is unknown whether this is ‘real’ or just due to which subjects remained and had data at 18 months.
